# Uptake of Modern Contraceptive Methods among Burundian Women and Associated Factors: Analysis of Demographic and Health Survey Data, Burundi 2016–2017

**DOI:** 10.24248/eahrj.v5i1.653

**Published:** 2021-06-11

**Authors:** Edouard Nkunzimana, Mu’awiyyah Sufiyan Babale, Adolphe Ndoreraho, Joseph Nyandwi

**Affiliations:** a Ministry of Public Health and Fight against AIDS, National Institute of Public Health, Bujumbura, Burundi; b Department of Community Medicine, College of Medical Sciences, Faculty of Clinical Sciences, Ahmadu Bello University, Zaria, Nigeria

## Abstract

**Background:**

Globally in 2017, Burundi was the 9^th^ country with the highest population growth rate of 3.2% and a fertility rate of 5.5 children per woman. This probably suggested low uptake of Modern Contraceptive methods (MCM) in the country. Our analysis investigated factors associated with low uptake of MCM among women of reproductive age in Burundi.

**Methods:**

Cross sectional data of non-pregnant women aged 15-49 years was extracted from the Burundi Demographic and Health Survey (2016-2017). We analysed the data at univariate, bivariate and multivariate levels to assess factors influencing MCM uptake among these women using Epi-Info 7.2.2.6.

**Results:**

Of the 9,945 women, 2,372 (23.8%) were using MCM. Ngozi province had the highest prevalence of MCM users [284/691(37.7%)]. The most used MCM among respondents was injectable contraceptive (48.3%). As respondent's age increases, the odds of using MCM decreases; 20-24 years (aOR=0.9, 95% CI [0.6–1.2]), 30–34 years (aOR=0.8, 95% CI [0.5–1.0]), 35-39 years (aOR=0.7, 95% CI [0.5–0.9]), 40-44 years (aOR=0.5, 95% CI [0.5–0.9]) and 45-49 years (aOR=0.4, 95% CI [0.2–0.5]) compared with those in the age group 15–19 years. Muslims (aOR=1.5, 95% CI [1.2–1.9]) and Jehovah witnesses (aOR=3.1, 95% CI [1.7–6.5]) were more likely to use MCM than Catholics.

**Conclusion:**

The prevalence of MCM remains low among women of reproductive age in Burundi, with injectables being the most used method. Factors such as respondent's age and religion were significantly associated with MCM use. Enhanced access to family planning information and services targeting women who are 30 years or more and engaging religious leaders for their active participation is recommended.

## BACKGROUND

In 1900, the world population was estimated to be about 1.7 billion.^1^ By 2018, the population had risen to 7.6 billion.^2^ Sixty percent (60.0%) of the world's population live in Asia (4.5 billion), 17.0% in Africa (1.3 billion), 10.0% in Europe (742 million), 9.0% in Latin America and the Caribbean (646 million), the remaining 6.0% in Northern America (361 million) and Oceania (41 million).^2^ China (1.4 billion) and India (1.3 billion) remain the 2 most populous countries of the world, comprising 19.0% and 18.0% of the global total, respectively.^2^ Currently, 80.0% of the world's population resides in less developed countries and this figure is expected to reach 90.0% by 2050.^1^

The world's population continue to grow albeit more slowly than in the recent past. Ten (10) years ago, the global population was growing by 1.2% per year. Today, it is growing by 1.1% per year, yielding an additional 83 million people annually.^2^ The European Union (EU) population is now growing slowly and is even expected to decline further in long term. So, the EU represents an ever-shrinking proportion of the world's population, at just 6.9% today down from 13.5% in 1960, and is projected to decline further by the end of this century to just 4.1%.^3^ Rapid population growth in Africa is anticipated even though there assumptions that there will be a substantial reduction of fertility levels in the near future. The medium-variant projection assumes fertility in Africa will fall from around 4.7 births per woman in 2010-2015 to 3.1 in 2045–2050, reaching a level slightly above 2.1 in 2095-2100.^2^

Burundi is among the 10 countries with highest fertility rates globally by 2017 (occupying the 9^th^ position), with 5.5 children per woman.^4^ Historical data shows that uptake of modern contraceptive method grew from 16.9% to 18.8% in 2012 and 2016 respectively.^5^ In terms of maternal health, maternal death rate has decreased from 1,220 (1990) to 712 (2015) per 100,000 live births.^6^ However, it is still among countries classified by the World Health Organisation (WHO) as having made no progress towards reducing Maternal Mortality Ratio (MMR) between 1990 and 2015.^7^ Studies have shown that unintended pregnancies among young women greatly contribute to high maternal and neonatal mortality through increased risk of unsafe abortion, birth injuries and postpartum depression.^8,9^

Therefore, delaying or avoiding pregnancies among young women and reducing the number of pregnancies among older women are key interventions in preventing and reducing maternal deaths more especially in countries with high maternal mortality like Burundi.

Universal access to effective contraceptive methods ensures that all adults and adolescents can avoid the adverse health and socio - economic consequences of unintended pregnancy while living a satisfying sexual life. Key global initiatives; the Sustainable Development Goals and the Global Strategy for Women's, Children's and Adolescent's Health call for universal access to family planning services as a right for women and girls, this is crucial to a healthy life.^10^ Women, men, or couples can choose from the many available contraceptive methods to help them plan their family and prevent unplanned pregnancy. They also need to know that, during the next 12 months, if they are having sex regularly and do not use any contraceptive method, about 8 out of every 10 women will become pregnant.^11^

Despite efforts taken by the Government of Burundi in favour of family planning which helped to increase the prevalence rate of modern contraceptives from 2.7% in 2000 to 34.0% in 2014^12^; the country, in 2017, remains in the ninth position globally in terms of high growth (3.2%) and fertility rate (5.5 children per woman).^5^ The Burundi general population was projected to be 11,890,784 in 2021, making a density of 463 inhabitants/km^2^.^13^ The determinants of modern contraceptive up-take have been explored around the world among women of child bearing age (15–49 years).^8,14–19^ For instance, in northwest Ethiopia, in 2015, Modern contraceptive methods utilisation was found to be 31.7%.^20^ In the same period, its prevalence in Dibindi, Democratic Republic of Congo (DRC) was 18.4%.^16^ Factors like age, education status, marital status, Spousal announcement about family planning issues, residence and income among others were factors associated with modern contraceptive methods utilisation.^15,20–22^ However, published data on these factors among Burundian women is limited. Understanding the key factors influencing modern contraceptive uptake among women of reproductive age who are at a higher risk of maternal morbidity and mortality will help inform appropriate interventions that could improve up-take of modern contraceptives. Therefore, the aim of this analysis is to investigate the factors associated with low uptake of MCM among women of reproductive age in Burundi.

## METHODS

### Study Design and Data Source

A secondary analysis of cross-sectional household data for women of reproductive age collected during the 2016-2017 Burundi Demographic and Health Survey was conducted. The survey was aimed at producing representative results at country level, urban and rural area level, Bujumbura city and other provincial levels. In achieving this, the national territory was divided into 18 fields of study corresponding to the 18 provinces and in each field of study (except Bujumbura Mairie which has no rural part), 2 strata were created: the urban and the rural stratum, from where samples were drawn.

2 stage sampling technique was used in this study; In the first stage, 554 Primary Sampling Units (PSU) or clusters were drawn from a list of Enumeration Areas (EAs) established during the 2008 General Population and Housing Census (RGPH), using systematic sampling technique with allocation proportionate to size. The size of the PSU is the total number of households in that particular unit. A list of households in each of the PSU/clusters provided the sampling frame in which 30 households per cluster were drawn using systematic sampling technique, also in the second stage from both urban and rural areas. A total of 16,637 households (3,191 in urban areas from 106 clusters and 13,446 in rural areas from 448 clusters) were selected. All women aged 15–49 years, usually living in the selected households, or present the night before were eligible for the survey. 4 questionnaires were used to collect data. Total number of eligible women interviewed was 17,269; among them, women who were not pregnant were asked if they ever used any method to avoid getting pregnant. In our analysis, only participants who provided responses to all the variables (dependent - contraceptive uptake and independent - religion, education, age, and others) were included. Hence, the sample size that was finally used for the analysis was 9,945.

### Study Variables and Measurements Dependent Variable

The outcome variable in this study was modern contraceptive methods uptake. Women who reported current use of modern contraceptive methods were considered as current users of modern contraceptives and those who responded that they use traditional methods or with a ‘no’ answer were regarded as non-users.

### Independent Variable

The independent variables included socio-demographic information such as age (15-19, 20-24, 25-29, 30-34, 35-39, 40-44 and 45-49 years), religion (Catholic, Protestant, Muslim, Jehovah witness, Adventist and no religion), socio-economic information which included the type of residence (urban and rural), education (none, primary school, secondary school or above), ownership of radio or television, and work/employment (working or not working). Other independent variable included was breastfeeding status.

### Data Management and Analysis

We downloaded data from the Burundi Demographic and Health Survey (DHS) program which was transferred into Microsoft Excel 2016 for cleaning. The data was then transferred into Epi-Info 7.2.2.6 for re-coding and analysis of the variables to suit the study objectives. Descriptive statistics was used to summarise the data and results were presented as frequency and proportions in tables and charts. Bivariate analysis (Pearson Chi square [x^2^]) was conducted to determine the association between modern contraceptive uptake and each of the predictor variables. Variables that had an association with modern contraceptive uptake at ≤0.2 at bivariate level were further analysed using unconditional multiple logistic regression to identify independent predictors of modern contraceptive uptake. Crude and Adjusted Odds Ratios and their 95% Confidence Intervals (95% CI) were estimated. All statistical analyses were performed at statistical significance level of *P-value* equals or less than .05. Quantum Geographic Information System Version 3.0.2 (QGIS 3.0.2) was used to draw the map of the study location.

## RESULTS

### Socio-Demographic Characteristics

A total of 9,945 women were eligible for the analysis based on the set inclusion criteria. The respondents’ mean age was 33.1±8.2 years. Majority, 5709 (57.4%) of the respondents were within 25 to 39 years’ age group. Most of them, 8,069 (81.1%) were residing in rural areas. Overall, Catholics were the majority 5,681 (57.1%) followed by protestants [3,361 (33.8%)], while Muslims were 435 (4.4%) and Jehovah witness were 33 (0.3%) among the respondents. About half [4,695 (47.2%)] of the respondents had no formal education, those with primary education were 3,849 (38.7%) while those who had secondary or more were 1,401 (14.1%). More than half [5,638 (56.7%)] were breastfeeding. In terms of employment status, 8,666 (87.1%) were working, less than half of the respondents [4,048 (40.7%)] owned either a radio or television among which only 1,735 (17.5%) were listening to radio or watching television at least once a week.

### Utilisation of Modern Contraceptive Methods

Out of the 9,945 respondents, 2,378 (23.9%) were using modern contraceptive methods at the time of interview. Among those who were using modern contraceptive methods, 1,146 (48.2%) were using injectables, followed by implants accounting for 627 (26.43%) of the respondents. Pills and male condom were used by 180 (7.6%) and 174 (7.3%) respondents respectively. The least used methods were Emergency contraception [9 (0.4%)] and female condom [2 (0.1%)] respectively (Table 1).

**TABLE 1:** Distribution of modern contraceptive users and type of contraceptives used, Burundi, 2016–2017

Characteristics	Frequency	Percent
**Modern contraceptive method use**		
Users	2372	23.85
No users	7573	76.15
**Type of methods used**		
(n= 2372)		
Injections	1146	48.31
Implants/Norplant	627	26.43
Pill	180	7.59
Male condom	174	7.34
IUD	96	4.05
Female sterilisation	60	2.53
Standard days method (SDM)	47	1.98
Lactationalamenorrhea (LAM)	19	0.80
Male sterilisation	12	0.51
Emergency contraception	9	0.38
Female condom	2	0.08

**FIGURE 1:**
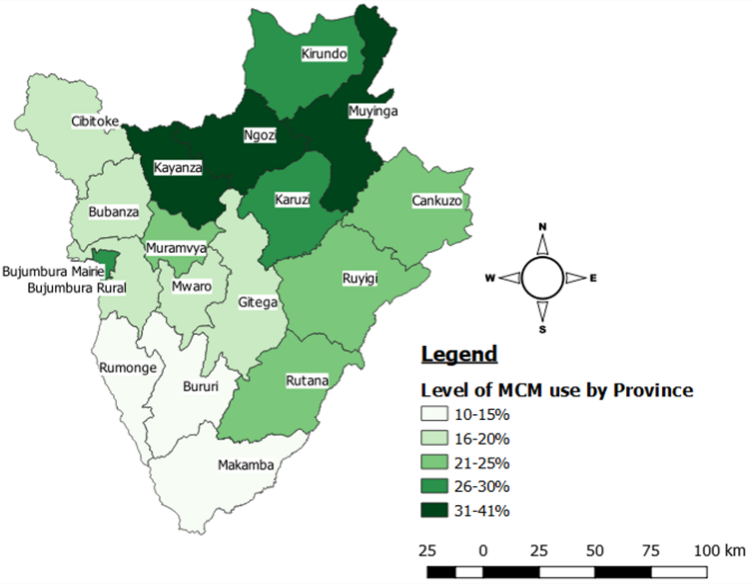
Prevalence of MCM use by province

Figure 1 shows that the prevalence of MCM uptake varies from one province to another. It was highest in the Northern part of the country; Ngozi province (41.1%) followed by Kayanza (34.9%) and Muyinga (31.6%) province. The provinces in the South had the lowest prevalence of 14.9%, 13.9% and 12.5% for Rumonge, Bururi and Makamba province respectively.

**FIGURE 2:**
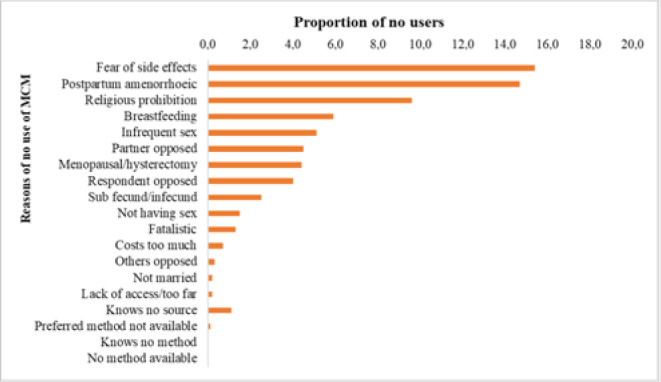
Reasons of not using MCM, Burundi, 2017

Among those who were not using MCM at the time of interview, One-fifth (20.9%) of them mentioned postpartum amenorrhoea as the reason for not using MCM. Other reasons given were; fear of side effects (12.9%), religious prohibition (7.7%) and having infrequent sex (4.9%). The reason with least responses was the non-availability of the method/service (Figure 2).

### Factors Associated with Modern Contraceptive Uptake

Table 2 shows that background characteristics of women are associated with the uptake of MCM. After controlling the possible confounders, the results from unconditional logistic regression in table 3 shows that women aged 30–34 years (aOR = 0.7; 95% CI = 0.5–0.9), 35–39 years (aOR=0.7; 95% CI=0.5–0.9), 40–44 years (aOR=0.6; 95% CI=0.4–0.8) and 45–49 years (aOR=0.4; 95% CI=0.2–0.5) were less likely to use modern contraceptive methods compared with young adolescent women aged 15–19 years. Being an urban resident was also significantly associated with modern contraceptive methods uptake (aOR=1.3; 95% CI=1.2–1.5). Compared with Catholics, Protestants (aOR=0.8; 95% CI=0.7–0.8) and those without religion (aOR=0.6; 95% CI=0.4–0.9) were less likely to use modern contraceptive methods. However, Muslims (aOR=1.5, 95% CI=1.2–1.9) and Jehovah witness (aOR=3.1; 95% CI=1.6–5.9) were more likely to be users of modern contraceptive methods. Having primary education (aOR=1.2; 95% CI=1.1–1.3) or secondary/higher education (aOR=1.2; 95% CI= 1.1–1.6) was not significantly associated with modern contraceptive methods uptake compared with those without any formal education (Table 3).

**TABLE 2:** Association between respondents' background characteristics and modern contraceptive use Burundi, 2017

Characteristics	Modern contraceptive methods use					
	Yes Freq	%	No Freq	%	χ^2^	*P-value*
**Age**						
15–19	67	31.0	149	68.9	211.4	<.001^*^
20–24	455	31.2	1004	68.8		
25–29	566	27.4	1499	72.6		
30–34	513	25.9	1466	74.1		
35–39	398	23.9	1267	76.1		
40–44	254	18.1	1149	81.9		
45–49	119	10.3	1039	89.7		
**Residence**						
Rural	1784	22.1	6285	77.9	70.9	<.001^*^
Urban	588	31.3	1288	68.7		
**Religion**						
Catholic	1393	24.5	4288	75.5	110.2	<.001^*^
Protestant	694	20.6	2667	79.3		
Muslim	178	40.9	257	59.1		
Adventist	70	23.3	230	76.7		
Jehovahwitness	17	51.5	16	48.5		
No religion	20	14.8	115	85.2		
**Highest educational level**						
No education	900	19.2	3795	80.8	118.8	<.001^*^
Primary	1033	26.8	2816	73.2		
Secondary/higher	439	31.3	962	68.7		
**Visited HF in the last 2 months**						
Yes	2137	24.8	6471	75.2	33.1	<.001^*^
No	235	17.6	1102	82.4		
**Currently working**						
Yes	1376	17.1	6659	82.9	17.5	<.001^*^
No	996	52.2	914	47.8		
**Having Radio/TV**						
Yes	1151	28.4	2897	71.6	78.5	<.001^*^
No	1221	20.7	4676	79.3		
**Listening Radio/TV**						
Not at all	1072	20.7	4096	79.3	69.3	<.001^*^
Less than once a week	423	24.4	1312	75.6		
At least once a week	877	28.8	2165	71.2		
**Currently breast feeding**						
Yes	1376	24.4	4262	75.6	2.1	.144
No	996	23.1	3311	76.9		

* Statistically significant

**TABLE 3:** Logistic Regression for Independent Predictors of Modern Contraceptive Use

Characteristics	cOR	95% CI	*P-value*	aOR	95% CI	*P-value*
**Age**						
15–19	Ref			Ref		
20–24	0.9	0.7–1.3	.692	0.9	0.6–1.2	.439
25–29	0.8	0.6–1.1	.133	0.8	0.5–1.0	.085
30–34	0.7	0.5–2.0	.049^*^	0.7	0.5–0.9	.023^*^
35–39	0.7	0.5–0.9	.025^*^	0.7	0.5–0.9	.019^*^
40–44	0.5	0.4–0.7	<.001^*^	0.6	0.4–0.8	.002^*^
45–49	0.3	0.2–0.4	<.001^*^	0.4	0.2–0.5	<.001^*^
**Residence**						
Rural	Ref			Ref		
Urban	1.6	1.5–1.8	<.001^*^	1.3	1.2–1.5	<.001^*^
**Religion**						
Catholic	Ref			Ref		
Protestant	0.8	0.7–0.9	<.001^*^	0.8	0.7–0.8	<.001^*^
Muslim	2.1	1.7–2.5	<.001^*^	1.5	1.2–1.9	.002^*^
Adventist	0.9	0.7–1.2	.646	0.8	0.6–1.1	.170
Jehovah witness	3.0	1.5–5.8	.001^*^	3.1	1.6–5.9	.001^*^
No religion	0.5	0.3–0.9	.015^*^	0.6	0.4–0.9	.041^*^
**Highest educational level**						
No education	Ref			Ref		
Primary	1.5	1.34–1.7	<.001^*^	1.2	1.1–1.3	.055
Secondary/higher	1.9	1.7–2.2	<.001^*^	1.2	1.1–1.6	.082
**Visited HF in last 2 months**						
No	Ref					
Yes	1.4	1.2–1.6	<.001^*^	1.1	1.0–1.3	.104
**Currently working**						
No	Ref					
Yes	0.8	0.7–0.9	<.001^*^	0.9	0.8–1.0	.069
**Having radio/TV**						
No	Ref					
Yes	1.5	1.4–1.6	<.001^*^	1.2	1.1–1.4	.002^*^
**Listening Radio/TV**						
Not at all	Ref			Ref		
Less than once a week	1.2	1.1–1.4	.009^*^	1.1	1.0–1.3	.068
At least once a week	1.5	1.4–1.7	<.001^*^	1.2	1.1–1.4	.066
**Currently breast feeding**						
No	Ref					
Yes	1.4	1.3–1.5	<.001^*^	1.1	1.0–1.2	.034^*^

* Statically significant

## DISCUSSION

The results of the analysis indicated that the prevalence of MCM is low among women of reproductive age in Burundi. Despite the effort of the Burundi government and the ministry of health in mobilising people on modern contraceptive uptake, utilisation has dropped from 30.8% in 2013^12^ to 23.8% in 2017. This can be explained by the non-availability of law that limits the number of children a woman should have. The prevalence in Ngozi province was the highest in the country and above the national average level. It is also the second province with highest population density^23^. This could probably explain their high uptake of MCM since they do understand the consequences of having many children and therefore agree to embrace family planning/child spacing services. The prevalence of MCM from this analysis is similar to that reported in the Burundi Demographic and Health Survey in 2016–2017 where 23.0% of non-pregnant women were using MCM as against 6.0% who were using traditional methods.^24^ Our findings were also consistent with that of Aviisah et al in Ghana (2014) and those from various studies in Africa, where the prevalence was 21.5%^18^ and between 20.0% and 30.0%.^15,16,18,20–22^ However, the uptake of MCM in Burundi is lower than uptake in Malawi where the prevalence was 30.9% in 2016.^8^ In a study conducted in northwest Ethiopia, uptake was also found to be higher than that of Burundi where 44.6% of women always used one of the MCM^20^. The most used methods among women were injections/injectables and implants/norplant. This finding was consistent with that obtained by Alemayehu et al in Ethiopia where injections and implants were the most used contraceptive methods.^21,22^ However, our results differ from the study conducted in Kenya where the commonly used contraceptives were condoms (35%) and pills (33%).^19^

Most of the respondents’ socio-demographic characteristics were significant predictors of MCM uptake. We observed that young women (aged 15-24 years) were more likely to use MCM compared to older women. This however, differs from the results of Ntambue et al in Mbuji-Mayi, DRC in 2015 where the age was not significantly associated with the uptake of MCM.^16^ Our analysis in the final adjusted model revealed that educational level of a woman did not influence her likelihood to use modern contraceptives. This differ from what Aviisah et al found in Ghana (2014), where women who had primary education as their highest level of educational attainment were 27.0% more likely to use modern contraceptives than women who had no formal education. While those who attained higher educational levels were 48.0% more likely to use modern contraceptives than women without formal education.^18^ A study conducted in Bangladesh on prevalence and determinants of contraceptive use among employed and unemployed women revealed that employed women with higher educational levels had a marked increased probability of contraceptive use compared to illiterates.^25^ Place of residence to a large extent, by default, influence different living habit. It was found that uptake of modern contraceptives was 33.0% higher among women in urban areas than those in rural areas. This is consistent with what was found in Ghana (2014).^18^ Religion play a major role in family planning methods utilisation. Religious beliefs about modern family planning varies from one region or faith to the another. Our analysis found that Protestants were 25.0% less likely to use MCM than Catholics. Muslims were 51.0% more likely to use MCM than other faith. Jehovah witness was also 3 times more likely to use MCM when compared with Catholics. Okech et al also found that religion negatively affects the uptake of MCM in Kenya's City Slums.^19^ However, a study in Ghana (2014)^18^ found no statistically significant association between religion and MCM use. Having radio or television was also found to be among the predictors of MCM use in our study, which is consistent with the study conducted in South Ethiopia by Tadesse et al in 2013.^21^

## CONCLUSION AND RECOMMENDATIONS

The prevalence of MCM in 2017 among women of reproductive age in Burundi was low and has a declining trend compared with what was obtained in 2013. Place of residence, religion and having a radio/television are significant predictors of MCM use among women in Burundi. In order to improve utilisation of modern contraceptive methods among women of reproductive age in Burundi, we recommend the following:

– Family planning program officers should strengthen family planning education especially among the rural residents.
– Health professionals should engage religious leaders in the promotion of family planning services to their respective congregations in order to do away with religious beliefs that act as barriers to MCM use.

### Limitations of the Study

This study relied on reported rather than actual MCM uptake. Future studies should consider prospectively or transversally collecting data within a specified period. However, this was beyond the scope of this study.

### Ethical approval and consent to participate

Permission to use the data was obtained from the DHS program. The original study obtained ethical clearance from the Burundi National Ethics Committee (NEC). All participants provided oral informed consent.
